# The Family Health Strategy and new horizons for the quality of primary care in Brazil

**DOI:** 10.1590/0102-311XEN051226

**Published:** 2026-06-22

**Authors:** Claunara Schilling Mendonça, Ana Luiza Ferreira Rodrigues Caldas

**Affiliations:** 1 Hospital de Clínicas de Porto Alegre, Universidade Federal do Rio Grande do Sul, Porto Alegre, Brasil.; 2 Ministério da Saúde, Brasília, Brasil.

It is an honor to accept the invitation to comment on the article *Thirty Years of the Family Health Strategy in the Brazilian Unified National Health System: Milestones, Advances, Setbacks and Challenges* ^1^, which is presented in such a comprehensive and thought-provoking manner, as is characteristic of its authors. Not only because the strategy is the largest social inclusion initiative within the Brazilian Unified National Heath System (SUS, acronym in Portuguese) but also because it has been an integral part of our personal and professional journey from its inception to the present day. The text highlights the major milestones of a program that has evolved into policy, the advances and setbacks over the last 30 years and the funding, workforce and care coordination challenges faced by the health care system.

Funded by the Federal Government through the Primary Health Care Floor, which introduced the transfer of federal funds directly to municipal health funds, and as a result of a three-tiered governance structure, this initiative is the cornerstone of Brazil’s primary care model. The number of family health teams, community health workers, oral health services and Family Health Expanded Centers (NASF, acronym in Portuguese) - now called multidisciplinary teams in primary health care - has grown considerably. Priority has been given to equity, with special incentives to serve historically neglected populations, such as quilombos, agrarian reform settlements, indigenous communities, riverine communities, incarcerated people and the homeless.

The alignment of family health with other social policies - such as the work-based health education Education Program for Work in Health (PET Saúde, acronym in Portuguese), the School Health Program and the More Doctors Program in coordination with the Ministry of Education ^2^ ^,^ ^3^ ^,^ ^4^, and the Brazilian Income Transfer Porgram cash transfer program in collaboration with the Brazilian Ministry of Development and Social Assistance, Family and Fight against Hunger - are some examples. However, an effective primary health care policy also enables coordination across the health sector, as is the case with home care and mental health services ^5^ ^,^ ^6^.

The challenges highlighted in the article - funding, workforce and care coordination across the wider health care network - have persisted over the past 30 years. While family health is once again a Federal Government priority and almost 30% of federal health care expenditure goes to primary health care (PHC), 80% of Brazil’s municipalities have a population of less than 20,000 inhabitants, and without technical and financial support from state departments of health, they are unable to ensure adequate patient flow through the intermunicipal service network. Apart from funding, the provision of quality longitudinal care requires a review of health team population density parameters. The parameters (3,500 people per team) set out in the current Brazilian National Primary Health Care Policy (PNAB, acronym in Portuguese) are often inconsistent with the epidemiological and social complexity of health territories. It is therefore imperative that national policy promotes technical and scientific studies to define new catchment parameters, possibly reducing this ceiling to enable family and community medicine specialists and multidisciplinary teams to provide effective expanded clinical care and manage care without being overburdened, which undermines the capacity to address health needs and deliver effective solutions.

During the first two decades of the Family Health Strategy (FHS), the primary focus was expansion to ensure coverage of the entire population through primary health care centers equipped with adequate facilities and human resources. In the third decade, the focus was the provision of quality care, which is not possible without specialists and a workforce that is adequately valued. In this respect, we face the risk of the commodification of care and the commercialization of health actions and services, with a shift from public management towards private provision through associations and outsourcing ^7^. With regard to the health workforce, the 40-hour workweek initially proposed in the PNAB should be thoroughly debated in light of the proposed constitutional amendment that seeks to introduce a workweek of up to 36 hours. The latter should be incorporated into current policy, adopting payments and benefits based on criteria that value the work of PHC workers, which is essential in a national policy ^8^.

The most complex aspect of building strong health care systems is the coordination of points of service delivery. Effective coordination of care through the integration and organization of the network starting with PHC requires strong management mechanisms; however, such mechanisms remain limited in Brazil, even in state capitals and larger municipalities. Recent specialized care policy, the review of scheduling policy and co-management of waiting lists alone cannot guarantee direct authorization of slots, effective priority setting and adequate response times, resulting in an imbalance in the coordination of PHC, even in regions that have scheduling centers or where appointments are scheduled by Family and Community Medicine. Against this backdrop of fragmentation, digital health and telehealth need to be incorporated into services not as support tools but as core elements for closing gaps in care, especially in small municipalities and remote areas. Telehealth enables real-time matrix support by enabling interprofessional consultation and reducing the distance between PHC and specialized outpatient care. Digital data integration is the path to addressing scheduling asymmetries, transforming electronic health records into a dynamic network co-management tool, with PHC playing a technological role as coordinator of patient flow ^9^ ^,^ ^10^.

Finally, PHC services are settings that enable the recognition, welcoming and appreciation of the multiple dimensions of human diversity, including biopsychosocial, socioeconomic, ethnic/racial, gender, sexual orientation, environmental, cultural and religious aspects. Effective community-based primary care is capable of guiding Brazilian society in the definition of needs and rights, incorporating the concepts of empowerment and social capital.

## Data Availability

Not applicable.
