# Supplementary material for: The Family Health Strategy and new horizons for the quality of primary care in Brazil
Source: Cad Saude Publica. 2026 Jun 22;42:e00051226. doi: 10.1590/0102-311XEN051226 (PMC13286393; doi:10.1590/0102-311XEN051226)
Supplement: Translation

# Translation: Estratégia Saúde da Família e os novos horizontes para a qualidade da atenção primária brasileira

**DOI:** 10.1590/0102-311XPT051226

**Authors:** Claunara Schilling Mendonça, Ana Luiza Ferreira Rodrigues Caldas

**Affiliations:** 1 Hospital de Clínicas de Porto Alegre, Universidade Federal do Rio Grande do Sul, Porto Alegre, Brasil.; 2 Ministério da Saúde, Brasília, Brasil.

Atender o convite de comentar o texto *30 anos da Estratégia Saúde da Família no Sistema Único de Saúde: Marcos, Avanços, Retrocessos e Desafios* ^1^, apresentado de forma muito abrangente e instigante, como é característico de seus autores, é uma honra, porque esta que é a maior política de inclusão social no Sistema Único de Saúde (SUS), é a parte da nossa trajetória pessoal e profissional desde a sua criação até o momento. O texto traz os marcos referenciais significativos de um programa que se transforma em política, os avanços e retrocessos nesses 30 anos e os desafios do financiamento, da força de trabalho e da coordenação do cuidado na rede de atenção à saúde.

Induzido pelo financiamento federal, com o Piso de Atenção Básica, que inaugura repasses federais diretos aos fundos municipais de saúde e como resultado de um empenho tripartite, impulsiona o modelo brasileiro de atenção primária. Crescem as equipes de saúde da família, os agentes comunitários de saúde, a saúde bucal e os Núcleos 

Ampliados de Saúde da Família (NASF), agora equipes multiprofissionais complementares aos cuidados primários, e esses recursos consideram a equidade, com incentivos especiais para o atendimento às populações historicamente negligenciadas, como em territórios quilombolas, assentamentos agrários, populações indígenas, ribeirinhas, privadas de liberdade e em situação de rua.

O alinhamento da saúde da família com outras políticas sociais, como o Programa de Educação para o Trabalho para a Saúde (PET Saúde), o Saúde nas Escolas e o Programa Mais Médicos com o Ministério da Educação (MEC) ^2^ ^,^ ^3^ ^,^ ^4^, e o acompanhamento das famílias que recebem o Bolsa Família, junto ao Ministério do Desenvolvimento e Assistência Social, Família e Combate à Fome são alguns exemplos, mas ter uma política de atenção primária à saúde (APS) efetiva permite articulação também dentro do setor saúde, como é o caso da atenção domiciliar e da saúde mental ^5^ ^,^ ^6^.

Os desafios apontados no artigo: financiamento, força de trabalho e coordenação do cuidado na Rede de Atenção à Saúde, permanecem nesses 30 anos. Embora a saúde da família tenha voltado a ser prioridade do Governo Federal e os recursos federais da assistência estejam próximos dos 30% para a APS, 80% dos municípios brasileiros têm uma população inferior a 20 mil habitantes, e sem apoio técnico e financeiro das Secretarias de Estado da Saúde não conseguem garantir o fluxo adequado dos usuários na rede de serviços intermunicipais. Para além do financiamento, a garantia da qualidade do cuidado longitudinal exige que revisitemos os parâmetros de densidade populacional por equipe. A previsão de até 3.500 pessoas por equipe, estimada na Política Nacional de Atenção Básica (PNAB) vigente, mostra-se muitas vezes incompatível com a complexidade epidemiológica e social dos territórios brasileiros. Torna-se imperativo que a política nacional induza estudos técnicos e científicos para definir novos parâmetros de adscrição, possivelmente reduzindo esse teto para permitir que o especialista em Medicina de Família e Comunidade e a equipe multiprofissional exerçam, de fato, a clínica ampliada e a gestão do cuidado sem a sobrecarga que compromete a resolutividade do cuidado ofertado.

Nas duas primeiras décadas da Estratégia Saúde da Família (ESF), o grande esforço era garantir sua ampliação e cobertura para toda a população brasileira, em unidades básicas de saúde (UBS) com infraestrutura adequada, incluídos os recursos humanos. A terceira década exigiu a garantia de sua qualidade, e isto não é possível sem profissionais especialistas e com a valorização e o reconhecimento adequados. Aqui enfrentamos o risco da mercantilização, o empresariamento nas ações e serviços de saúde, com a atual substituição da administração direta para a gestão com associações privadas e a contratação de pessoas de forma terceirizada e suas derivações ^7^. Ainda em relação à força de trabalho, a carga horária de 40 horas semanais, proposta inicialmente na PNAB visando a garantir os atributos da APS na ESF, hoje, à luz da proposta de Emenda à Constituição que propõe a jornada de trabalho de até 36 horas semanais, deve ser amplamente debatida e incorporada na política atual, adotando pagamentos e valores agregados com base em critérios que valorizem o trabalho do profissional da APS, imprescindível numa política de abrangência nacional ^8^.

O processo mais complexo na construção dos sistemas de saúde é a articulação dos pontos de atenção. A coordenação do cuidado com a integração e ordenação da rede a partir da APS requer mecanismos de gestão ainda incipientes no Brasil, mesmo nas capitais e municípios maiores. A recente política da atenção especializada e a revisão da política de regulação não garantem sozinhas a autorização direta de vagas, de definição de prioridades e tempos-resposta, nem mesmo uma cogestão das listas, permanecendo uma assimetria no papel coordenador da APS, mesmo nas regiões onde contam com centrais de regulação ou a regulação do acesso exercida pelo Médico de Família e Comunidade. Nesse cenário de fragmentação, a saúde digital e a telessaúde devem ser incorporadas não como ferramentas acessórias, mas como eixos estruturantes para a superação de vazios assistenciais, especialmente nos municípios de pequeno porte e em áreas remotas. A Telessaúde permite o matriciamento em tempo real com a interconsulta, reduzindo as distâncias entre a APS e a atenção ambulatorial especializada (AAE). A integração digital de dados é o caminho para mitigar as assimetrias na regulação, transformando o prontuário eletrônico em uma ferramenta viva de cogestão da rede, onde a APS exerce, tecnologicamente, o seu papel de ordenadora do fluxo assistencial ^9^ ^,^ ^10^.

Por fim, a APS é o cenário que possibilita o reconhecimento, o acolhimento e a valorização das múltiplas dimensões da diversidade humana, como os aspectos biopsicossociais, socioeconômicos, étnico-raciais, de gênero, orientação sexual, ambientais, culturais e religiosos. A atenção primária resolutiva e de base territorial é capaz de conduzir a sociedade brasileira na definição das necessidades e direitos, incorporando o conceito de empoderamento e capital social.

## Data Availability

Não se aplica.
